# Metabolic Model of the Nitrogen-Fixing Obligate Aerobe Azotobacter vinelandii Predicts Its Adaptation to Oxygen Concentration and Metal Availability

**DOI:** 10.1128/mBio.02593-21

**Published:** 2021-12-14

**Authors:** Alexander B. Alleman, Florence Mus, John W. Peters

**Affiliations:** a Institute of Biological Chemistry, Washington State University, Pullman, Washington, USA; McMaster University

**Keywords:** aerobes, ammonia excretion, bioenergetics, diazotrophs, metabolic modeling, nitrogen fixation, respiration

## Abstract

There is considerable interest in promoting biological nitrogen fixation (BNF) as a mechanism to reduce the inputs of nitrogenous fertilizers in agriculture, but considerable fundamental knowledge gaps still need to be addressed. BNF is catalyzed by nitrogenase, which requires a large input of energy in the form of ATP and low potential electrons. Diazotrophs that respire aerobically have an advantage in meeting the ATP demands of BNF but face challenges in protecting nitrogenase from inactivation by oxygen. Here, we constructed a genome-scale metabolic model of the nitrogen-fixing bacterium Azotobacter vinelandii, which uses a complex respiratory protection mechanism to consume oxygen at a high rate to keep intracellular conditions microaerobic. Our model accurately predicts growth rate under high oxygen and substrate concentrations, consistent with a large electron flux directed to the respiratory protection mechanism. While a partially decoupled electron transport chain compensates for some of the energy imbalance under high-oxygen conditions, it does not account for all substrate intake, leading to increased maintenance rates. Interestingly, the respiratory protection mechanism is required for accurate predictions even when ammonia is supplemented during growth, suggesting that the respiratory protection mechanism might be a core principle of metabolism and not just used for nitrogenase protection. We have also shown that rearrangement of flux through the electron transport system allows A. vinelandii to adapt to different oxygen concentrations, metal availability, and genetic disruption, which cause an ammonia excretion phenotype. Accurately determining the energy balance in an aerobic nitrogen-fixing metabolic model is required for future engineering approaches.

## INTRODUCTION

In agriculture, nitrogenous fertilizers have become essential to maximizing crop yields to support the growing world population (1). Biological nitrogen fixation (BNF) is the reduction of atmospheric dinitrogen (N_2_) to ammonia (NH_3_) and accounts for ∼60% of the fixed nitrogen input into natural ecosystems (2). Nitrogenase, the enzyme catalyzing N_2_ reduction, is a significant energy sink, as it requires large amounts of ATP and low-potential electrons to produce NH_3_.

There are three types of nitrogenase, termed Mo-, V-, and Fe-only nitrogenases, reflecting the metal cofactor composition in the N_2_ reduction catalyst (3–5). Bacteria that contain V- and Fe-only nitrogenases are not dependent on Mo availability in the environment (6). Despite the similar features shared by the three nitrogenases, they differ in their reaction stoichiometry (7, 8). Whereas Mo-nitrogenase is the most efficient, requiring a minimum of 8 low-potential electrons and 16 MgATP to convert N_2_ to 2 NH_3_ *in vitro* (equation 1), the V- and Fe-only nitrogenases have lower catalytic activities and different reaction stoichiometries, requiring more electrons and ATP for catalysis and producing more H_2_ relative to NH_3_.

(1) $${\text{N}}_{2\;}+\;8e^{-}\;+\;\text{16MgATP}\;+\;{\text{8H}}^{+}\to 2{\text{NH}}_{3}\;+\;{\text{H}}_{2}\;+\;\text{16MgADP}\;+\;{\text{16P}}_{\text{i}}$$

Diazotrophs are physiologically diverse and include obligate aerobes, facultative anaerobes, anaerobic heterotrophs, anoxygenic or oxygenic phototrophs, and chemolithotrophs (9, 10). Under nitrogen-fixing conditions, diazotrophs must remodel their energy metabolism to provide nitrogenase with ATP and low-potential electrons while protecting the enzyme from irreversible inactivation by oxygen (11). Oxygen protection is not an issue for strict anaerobes; however, the ATP demands of nitrogen fixation during anaerobic metabolisms, such as fermentation, are profound relative to the ATP production per unit carbon (9). In contrast, oxygen respiration and the light reactions of photosynthesis can generate more energy for diazotrophic growth, but protecting nitrogenase from oxygen inactivation then becomes a larger consideration. Diazotrophs that live in the air protect nitrogenase from inactivation through various mechanisms that involve conditionally, temporally, or spatially separating oxidative phosphorylation or photosynthesis from nitrogen fixation (12).

The ubiquitous soil bacterium Azotobacter vinelandii is arguably one of the most robust and productive free-living nitrogen-fixing organisms (13, 14). A. vinelandii possesses a greater capacity to fix nitrogen than many other diazotrophs because of its ability to fix nitrogen at high oxygen concentrations (15). This ability is dependent on multiple mechanisms to protect nitrogenase from inactivation by oxygen (11, 16–18, 67). One of the primary mechanisms involves harnessing a robust and dynamic respiratory metabolism to balance the increased energy demands of nitrogen fixation while simultaneously consuming a significant amount of oxygen at the membrane. This process, termed respiratory protection, maintains high enough respiration rates to sustain low oxygen tensions in the cytoplasm (11). A branch of the electron transport chain increases oxygen consumption by partially decoupling ATP synthesis from O_2_ consumption (14, 19). To supply energy for respiratory protection, A. vinelandii catabolizes sugars through the Entner-Doudoroff and pentose phosphate pathways to produce acetyl coenzyme A (acetyl-CoA) and then predominately uses the tricarboxylic acid (TCA) cycle to deliver NADH (20, 21). During diazotrophic growth, A. vinelandii must efficiently balance the reduction of low-potential electron carriers, ATP production using oxidative phosphorylation, and protecting the nitrogenase enzyme from oxygen through the partially coupled branch of the electron transport chain.

**FIG 1:**
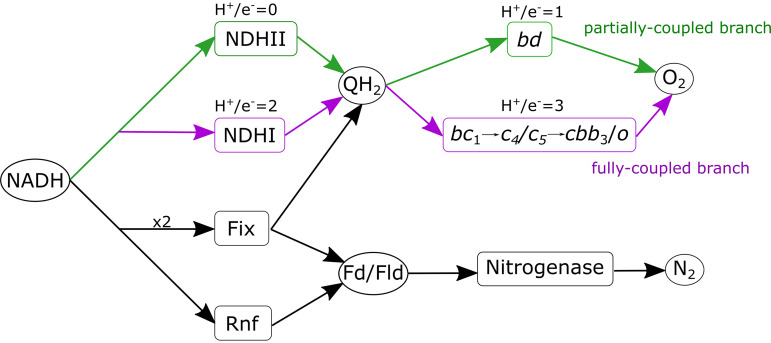
Paths of electrons in the electron transport system of A. vinelandii. Four different enzymes potentially consume NADH: uncoupled type II NADH dehydrogenase (NDHII), fully coupled NADH dehydrogenase (NDHI), flavin-based electron bifurcating Fix enzyme complex (Fix), and NADH:ferredoxin oxidoreductase (Rnf). There are two branches of the electron transport system (ETS) that perform oxygen reduction: (i) the partially coupled respiratory protection branch (green), beginning with NDHII reducing quinone to quinol (QH_2_) and terminating with cytochrome *bd*, and (ii) the fully coupled branch (purple), beginning with NDHI reducing quinone, in turn reducing cytochrome *c* and ending in cytochrome *o*-like terminal oxidase. Each branch translocates a different number of protons per electron, given above the reaction names. Production of reduced Fd/Fld occurs through enzyme complexes Fix and Rnf, which use NADH as the electron source. Nitrogenase consumes eight reduced Fd/Fld to reduce N_2_ to NH_4_^+^.

A. vinelandii adjusts respiration through a branched respiratory chain that includes multiple dehydrogenases and terminal oxidases. The chain’s two branches are classified as (i) the fully coupled branch and (ii) the partially coupled branch, respective to the protons translocated during oxidative phosphorylation (16) (Fig. 1). The branches of the respiratory chain are mediated by two distinct NADH:quinone redox reaction complexes (NDH). The first, NDHI, is coupled to the transmembrane proton potential and is mechanistically similar to complex I of mitochondria (22). However, the second, NDHII, is induced under high-aeration conditions and carries out NADH oxidation without translocating protons across the membrane, thus decoupling oxygen consumption from ATP generation (19). Other dehydrogenases (DHs) can also donate to the quinone pool, including malate DH, succinate DH, and hydrogenases. However, the first two of these DHs do not increase in expression under nitrogen-fixing conditions. However, H_2_ production is an obligatory part of the nitrogenase reaction (equation 1), and uptake hydrogenases recycle electrons from the H_2_ produced back into the quinone pool (23, 24).

The respiratory chain in A. vinelandii branches into two paths from the quinone pool, the fully coupled branch, including the *bc*_1_ complex, cytochrome *c*_4_/*c*_5_, and *o*-type or *cbb*_3_ terminal oxidases, and the partially coupled branch, with cytochrome *bd* (a quinol terminal oxidase). Cytochrome *bd* accumulates under high-aeration conditions, and knockout mutants lacking *bd* oxidase cannot grow diazotrophically at any aeration rate (13, 14, 25). The fully coupled respiratory branch terminates in a classical cytochrome *bc*_1_ reduction of cytochrome *c* to a terminal oxidase of cytochrome *o* or *cbb*_3_ (26). This branch has not been as well characterized in A. vinelandii. Still, kinetic evidence *in vivo* supports the existence of two cytochrome *c* terminal oxidases (13). Overall, the fully coupled branch consists of NDHI, cytochrome *bc*_1_, and cytochrome *o*/*cbb*_3_ translocating 10 H^+^ per ^1^/_2_O_2_ reduced, while the partially coupled branch consists of NDHII and cytochrome *bd* and only translocates 2H^+^ per ^1^/_2_O_2_ reduced (Fig. 1).

Under nitrogen-fixing conditions, A. vinelandii carbon catabolism directs most electrons to the reduction of NAD^+^, which has a reduction midpoint potential of ∼-320 mV, while nitrogenase requires electrons with a lower potential of ∼-500 mV (27). Thus, additional energy is required to transfer electrons from NADH to lower potential electron carriers, such as ferredoxin (Fd) or flavodoxin (Fld). Under nitrogen-fixing conditions, A. vinelandii expresses membrane-associated Fix and Rnf complexes that catalyze the endergonic reduction of Fd/Fld by NADH (28, 29). Rnf uses the proton motive force to provide the additional energy required in the reaction (30–32). The Fix complex uses flavin-based electron bifurcation in which it catalyzes the coordinated transfer of electrons from NADH to both quinone and Fd/Fld (29). The combination of branched electron transport to oxygen and the generation of reduced Fd/Fld through Fix and Rnf creates a complex and dynamic electron transport system (ETS) (Fig. 1).

The metabolic energy cost of nitrogen fixation in A. vinelandii was recently studied through carbon-based metabolomics (20) and investigated through multiple quantitative and metabolic models (33–35). However, these studies did not account for the dynamics of A. vinelandii’s ETS and its energy requirements; thus, they lack insights into enzyme pathways for energy homeostasis, or they fail to predict growth under high-oxygen and high-substrate conditions. By integrating A. vinelandii’s energy metabolism dynamics under nitrogen-fixing conditions into the genome-scale metabolic model and constraining the model with high-quality bioreactor data, an accurate growth and partitioning of resources can be predicted. Interestingly, the carbon cost of respiratory protection within the model is not entirely accounted for by decoupling energy through the partially coupled branch. The model also indicates that large amounts of energy are dedicated to maintaining respiratory protection even when fixed nitrogen is in the growth medium under laboratory conditions of high carbon and high oxygen concentrations. Understanding the distribution of flux throughout the ETS is essential in the development of ammonia-excreting diazotrophs. The energy requirements and the metabolic bottlenecks for newly engineered ammonia-excreting strains may be predicted with the model.

The future of agriculture is dependent on an affordable, renewable, and environmentally sound supply of nitrogenous fertilizer. Synthetic biology and BNF have the potential of alleviating some dependency on traditional fertilizing techniques. Nevertheless, an accurate understanding of nitrogen fixation at the systems level is required to maximize high-throughput synthetic biology abilities. The metabolic model presented here is the first step in understanding some of the dynamics of this complex system.

## RESULTS

### Curation of the metabolic model of A. vinelandii.

Recently, a metabolic model (iDT1278) was published that encompasses much of the A. vinelandii genome, establishing carbon and nitrogen sources using Biolog plate experiments (35). This framework for understanding the metabolism of A. vinelandii is a valuable tool for quantifying the production of biopolymers. However, the model iDT1278 lacked essential enzymes required for nitrogen fixation and failed to determine an accurate growth rate in standard laboratory conditions of complete aeration and at least 10 g/L of sucrose or equivalent carbon source (36) (Table 1). Therefore, a new model (iAA1300) presented here expands the iDT1278 model by adding missing reactions and manually curating inadequately annotated constraints (Table S1). Specifically, enzymes of the ETS were added to iAA1300, including Fix, NDHI, a quinone:cytochrome c oxidoreductase, V- and Fe-only nitrogenase, and a transhydrogenase, all of which have been biochemically or genetically determined to play a role during nitrogen fixation.

**TABLE 1:** Growth rates and physiological parameters are predicted from flux balance analysis results^*a*^

Model	Growth rate (h^−1^)	
	Ammonia supplemented	Diazotrophic
Exptl^*b*^	0.27	0.25
iDT1278	1.87	1.28
iAA1300		
No added constraints	1.43	0.98
ED and GS constrained	1.37	0.93
ED, GS, and maintenance constrained^*c*^	0.38	0.24

a All models have a glucose uptake rate of 15 mmol_glucose_ · h^−1^ · g CDW^−1^. ED, Entner-Doudoroff; GS, glyoxylate shunt.

b Data from the work of Wu et al. (20).

c ATP maintenance rate of 110 mmol ATP h^−1^ gCDW.

10.1128/mBio.02593-21.1 TABLE S1 Reactions added to the model iAA1300, with the common name, reaction stoichiometry, and gene reaction associations. Annotation terms for FIX are terms for electron transfer flavoproteins (ETFs), as the electron-bifurcating enzyme complex is not yet in databases. V-nitrogenase does have a KEGG annotation, but the stoichiometry is inaccurate. Fe-only nitrogenase has no annotation in any database. Download Table S1, XLSX file, 0.01 MB. Copyright © 2021 Alleman et al. 2021 Alleman et al. https://creativecommons.org/licenses/by/4.0/ This content is distributed under the terms of the Creative Commons Attribution 4.0 International license.

After manual curation, the model required central carbon metabolism constraints to represent experimental results more accurately. First, unlike other species in the family *Pseudomonadaceae*, A. vinelandii contains phosphofructose kinase and has a complete Embden-Meyerhoff pathway (37). Nevertheless, multiple studies have shown that A. vinelandii utilizes the Entner-Doudoroff (ED) pathway (20, 38). Flux into the ED pathway and the glyoxylate shunt was constrained to a ratio determined previously by ^13^C-metabolic flux analysis (20). While these constraints directed carbon into the correct pathways, the predicted growth rate for model iAA1300 was still inaccurate (Table 1).

### Establishing constraints for accurate growth rate determination.

Model iAA1300 overestimated growth in almost every condition due to the lack of accurate non-growth-associated maintenance flux (NGAM). Microbiologists since the 1950s have observed that the genus *Azotobacter* has an unusually high respiration rate, leading to increased maintenance requirements (68, 69). The high maintenance and respiration rate of A. vinelandii result in low biomass yields compared to other model proteobacteria. Quantitative modeling accurately described this phenomenon with large amounts of energy diverted to respiratory protection (33).

To translate the excess energy consumption into the genome-scale model, experimental data were used to predict an ATP maintenance (ATPM) rate under different O_2_ concentrations. Kuhla and Oelze (39) measured maintenance coefficients (mmol_substrate_ · h^−1^ · g CDW^−1^; “CDW” is dry weight of cells) of A. vinelandii growing in continuous diazotrophic cultures in different O_2_ concentrations and carbon sources, using the Pirt method (40) (Table 2). Maintenance coefficients increased as the O_2_ concentration increased in the bioreactor. Converting the experimentally determined maintenance coefficient to the genome-scale model ATPM reaction (mmol_ATP_ · h^−1^ · g CDW^−1^) requires an ATP/substrate ratio term. An issue arises with converting the maintenance coefficient to ATPM when considering the ATP produced per O_2_ consumed (P/O ratio) of the different branches of the ETS. The fully coupled branch uses 1 mol of glucose to produce 32 mol of ATP, but the partially coupled respiratory protection branch produces only 9 mol of ATP per mol of glucose. Experimentally during high substrate and high O_2_ conditions, A. vinelandii requires decoupling of the ETS through the partially coupled branch to maintain growth (25, 41, 42).

**TABLE 2:** Experimental measured maintenance and predicted ATP maintenance rate for both branches of the ETS^*a*^

Carbon source	[O_2_] (μM)	Maintenance coefficient (mmol_substrate_ · h^−1^ · gCDW^−1^)^*b*^	Predicted ATPM (mmol_ATP_ · h^−1^ · gCDW^−1^)	
			Fully coupled	Partially coupled
Sucrose	12	0.9	50.3	16.3
	48	4.4	245.6	78.8
	108	6.2	346.1	110.8
	144	7.0	390.9	125.0
	192	8.0	446.7	143.1
Glucose	108	14.8	364.4	111.4

a ATP maintenance rate was determined by setting substrate uptake rate to the maintenance coefficient and increasing ATP maintenance reaction flux until growth rate reached zero.

b Maintenance coefficient from the work of Kuhla and Oelze (39), converted from grams of protein to g CDW.

To confirm the use of the partially coupled branch, each path of the ETS network was tested to determine its accuracy to predict the growth rate. Two models were created, the first assuming that all flux to O_2_ is directed through NDHI and cytochrome *o* (fully coupled branch) and the second assuming all flux to O_2_ through NDHII and cytochrome *bd* (partially coupled branch) (Fig. 1). In both models, substrate uptake rates were set to the experimentally determined maintenance coefficients for each O_2_ concentration (39). This uptake rate represents the substrate consumption when no growth occurs; therefore, all energy produced must go to NGAM. To determine the corresponding NGAM for each condition, flux through the reaction ATPM was increased until the growth rate reached zero (Table 2).

**FIG 2:**
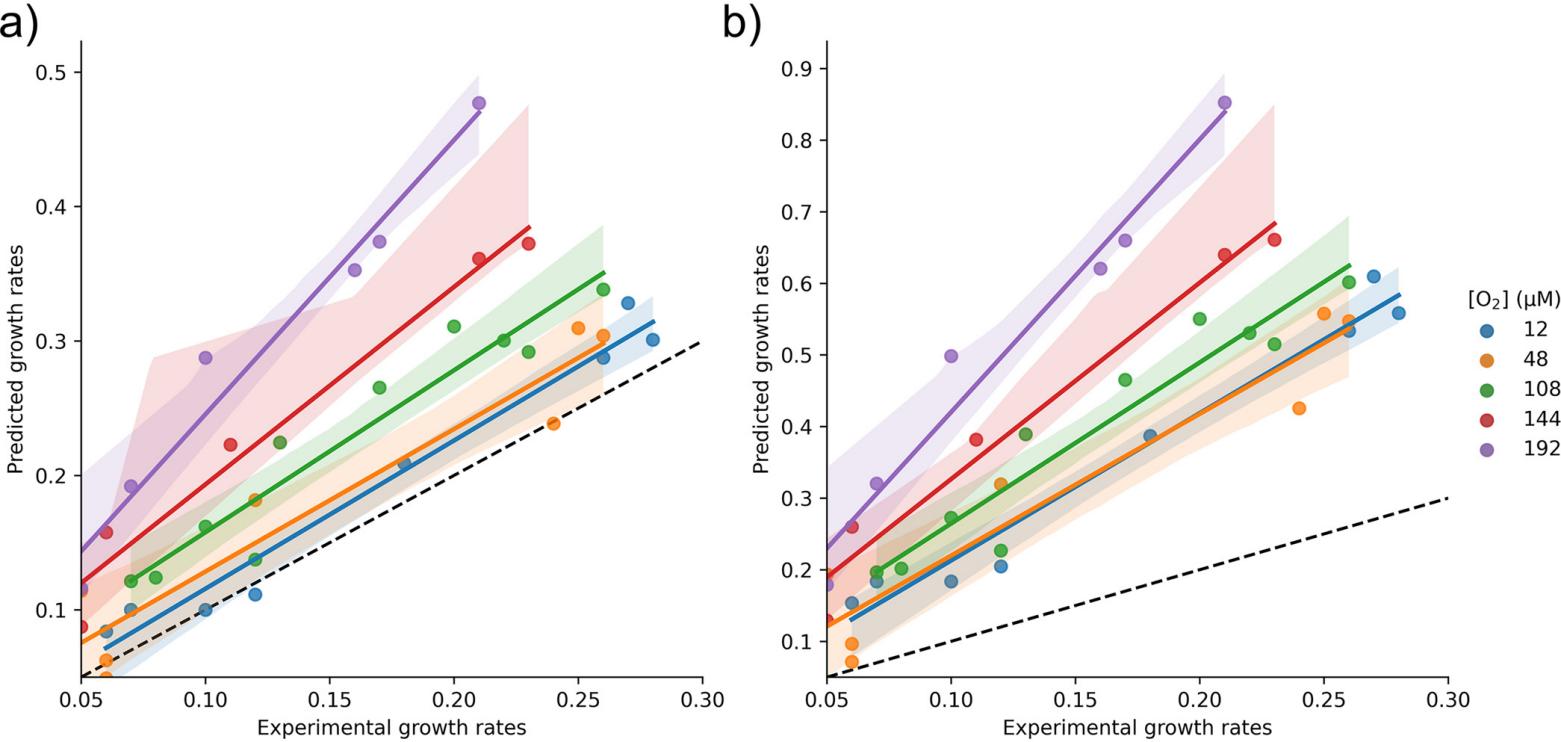
Each model was given an experimentally determined sucrose uptake rate, and a growth rate was predicted. Here, theoretical growth rate and experimental growth rate were compared for diazotrophic growth in different oxygen conditions. Perfect prediction rates follow the *x* = *y* dotted line. Shading represents the 95% confidence interval for linear regression fit. (a) Using the partially coupled branch to determine ATPM flux gives an accurate growth rate prediction for lower O_2_ concentrations of 12, 48, and 108 μM. Divergence from this trend occurs at 144 and 192 μM O_2,_ where the model overestimates growth. (b) Using the fully coupled branch of the ETS to determine the ATPM flux causes an overestimation of growth under all conditions.

The model-determined maintenance rates were then tested to predict growth rates at the different O_2_ concentrations. Each model was given an experimental substrate uptake rate and the predicted ATPM flux for each O_2_ concentration. Growth rates were then predicted and tested for error against experimental data. Using the fully coupled branch results in growth overestimates for all O_2_ concentrations (Fig. 2b). The partially coupled branch model predicted growth rates with a minor error, especially at lower O_2_ concentrations (Fig. 2a). The partially coupled model is within reasonable error across all growth rates for O_2_ concentrations of 12, 48, and 108 μM (Table S2). Adding these maintenance constraints to the model accurately predicts growth (Table 1). For the higher O_2_ concentration of 144 and 192 μM, the partially coupled branch model still overestimates growth. For these conditions, the respiratory protection and predicted maintenance could not account for the excess energy expenditure. Therefore, to better predict growth under high O_2_ concentration even with the partially coupled branch, more energy allocation to ATPM would limit biomass production, increase respiration, and slow predicted growth.

10.1128/mBio.02593-21.2 TABLE S2 Error of predicted growth rates compared to experimental growth rates for both ETS branches under different oxygen concentrations. MSE, mean square error; MAE, mean absolute error; RMSE, root mean squared error. Download Table S2, XLSX file, 0.01 MB. Copyright © 2021 Alleman et al. 2021 Alleman et al. https://creativecommons.org/licenses/by/4.0/ This content is distributed under the terms of the Creative Commons Attribution 4.0 International license.

### Assessment of growth yield in response to oxygen concentration.

**FIG 3:**
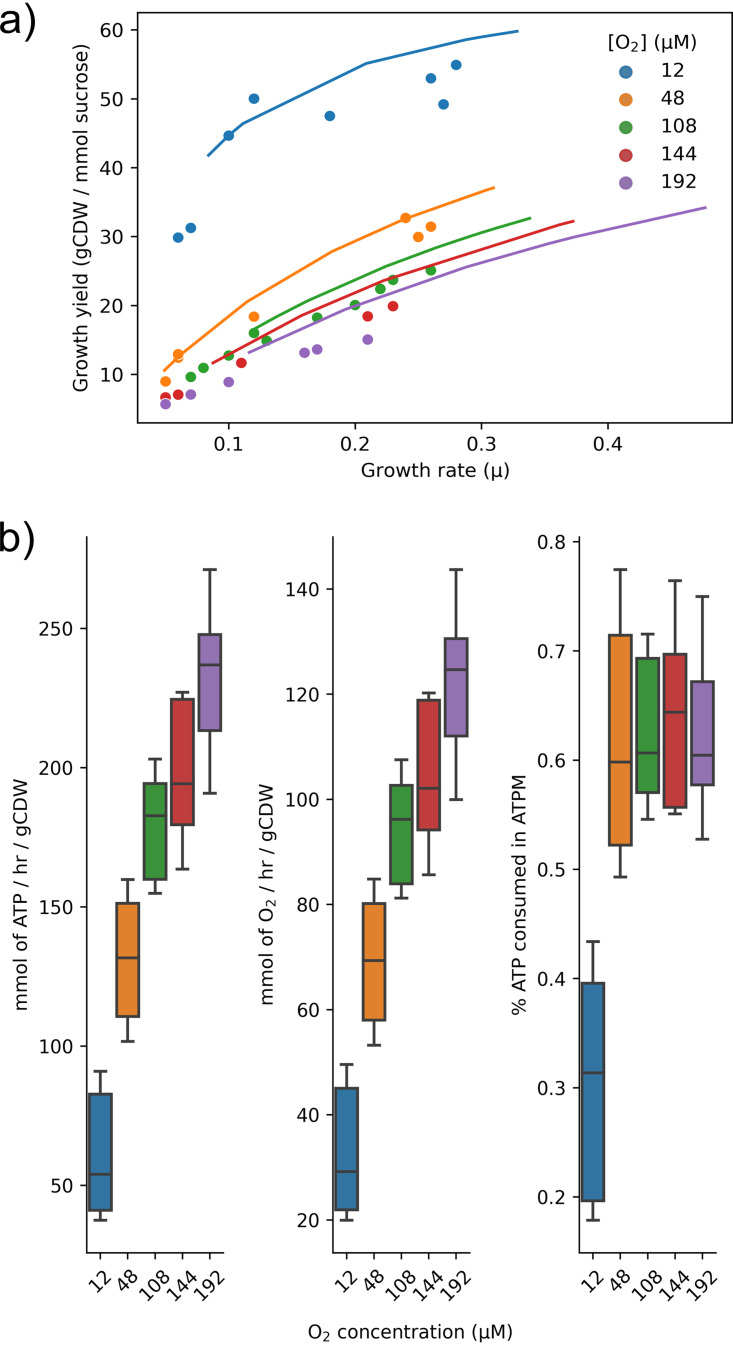
Allocation of carbon and energy at different O_2_ concentrations. (a) Predicted growth yields (lines) were plotted for each O_2_ concentration across multiple growth rates. Plotted versus experimental growth yields (points) show accurate predictions for lower oxygen concentrations. (b) Resource allocation across multiple O_2_ concentrations, sucrose uptake rates, and growth rates. Measured by flux through ATP synthase, respiration rates, and percentage of total ATP consumed in ATPM.

The overall growth efficiencies can be further investigated with detailed maintenance estimates. The growth yield was predicted using the experimental sucrose uptake rate and plotted versus the experimentally determined growth yield (Fig. 3a). Similar to the growth rate predictions, the growth yield predictions indicate that the 12, 48, and 108 μM O_2_ conditions are within error. The differentiation of growth yield between the 12 μM condition and higher-O_2_ conditions initially seen in the experimental data can be reproduced with the model. The original work of Kuhla and Oelze discussed this effect as the “decoupling of respiration” or respiratory protection (39). However, we have shown that the partially coupled branch is required even at 12 μM O_2_. To investigate this phenomenon in more detail, energy allocation during the increase of O_2_ concentration was plotted (Fig. 3b). The flux through ATP synthase and O_2_ respiration rates (cytochrome *bd*) increase linearly with O_2_ concentration. However, partitioning of the ATP differentiates the 12 μM condition from the high O_2_ concentrations. The percentage of ATP consumed in ATPM reaction plateaus at around 60% of total ATP consumed for 48, 108, 144, and 192 μM O_2_ but only at ∼30% for 12 μM O_2_. This differentiation allows more ATP to be utilized in biomass production, creating higher growth yields for the 12 μM O_2_ concentration.

### Ammonia-assimilating conditions require a high maintenance rate for accurate growth.

The respiratory protection mechanism was considered to be directly responsible for protecting nitrogenase from O_2_ damage (11). Experimentally, growth rates under ammonia-supplemented conditions are similar to those under diazotrophic conditions with high carbon, with comparable biomass yield (15, 20). Modeling ammonia-supplemented growth shows the requirement of the partially coupled branch to minimize ATPM and accurately predict growth (Table 1). While there is a lack of accurate physiological details on A. vinelandii grown in high-carbon and ammonia-supplemented medium, similar respiration rates have been reported (15). The energy allocation under ammonia-supplemented growth shows that the ATP not used in nitrogen fixation is utilized for biomass production (Fig. S1).

10.1128/mBio.02593-21.4 FIG S1 ATP allocation at high and low O_2_ concentrations and with or without ammonia supplementation. If the total flux of a subsystem was less than 1 mmol ATP · h^−1^ · g CDW^−1^, the subsystem was classified as “other.” Download FIG S1, TIF file, 1.8 MB. Copyright © 2021 Alleman et al. 2021 Alleman et al. https://creativecommons.org/licenses/by/4.0/ This content is distributed under the terms of the Creative Commons Attribution 4.0 International license.

### Effects of Rnf and Fix on accurate growth predictions.

While the model shows the requirement of the partially coupled branch to minimize flux through NGAM, little is known about Rnf and Fix’s roles under different O_2_ conditions. The use of Rnf or Fix was considered when the initial ATPM flux was determined, but neither path affected the overall cost of NGAM (Data Set S1). Under high-O_2_ conditions, the percent of electron flux required for Fd reduction is minimal compared to that for respiration. However, the different reaction mechanisms suggest that Rnf and Fix might play different roles within the ETS. This difference is accentuated when NDHII is used, as the energy from quinone reduction does not contribute to proton motive force. Therefore, in this scenario, with NDHII as the primary NADH oxidation site, the use of NADH to reduce Fd/Fld using Fix does not sacrifice translocated protons. Fix is favored as the flux increases to nitrogenase and away from O_2_ reduction, as it can maintain a higher ATP production rate (Fig. S2). Rnf consumes energy through the proton motive force, lowering growth yields compared to Fix, and increases O_2_ consumption, which could help predict high O_2_ concentrations more accurately.

10.1128/mBio.02593-21.5 FIG S2 Growth rate versus the ratio of flux to nitrogen reduction over flux to oxygen reduction. Models with deletions of genes encoding either Rnf or Fix were tested. As flux to nitrogenase is increased, the slope at which the growth rate declines is higher in models without Fix. Conversely, models without Rnf can sustain a higher growth rate as flux to nitrogenase is increased. Download FIG S2, TIF file, 2.1 MB. Copyright © 2021 Alleman et al. 2021 Alleman et al. https://creativecommons.org/licenses/by/4.0/ This content is distributed under the terms of the Creative Commons Attribution 4.0 International license.

10.1128/mBio.02593-21.7 DATA SET S1 ATP maintenance rates for all data points found in the work of Kuhla and Oelze (39) for each path in the ETS network to nitrogen and oxygen reduction. The experimental and predicted growth rates used in Fig. 2 are presented, as is predicted oxygen consumption rate. Predicted growth yields on sucrose and oxygen are calculated from the growth rates and sucrose or oxygen uptake rates. Download Data Set S1, XLSX file, 0.05 MB. Copyright © 2021 Alleman et al. 2021 Alleman et al. https://creativecommons.org/licenses/by/4.0/ This content is distributed under the terms of the Creative Commons Attribution 4.0 International license.

### Flux sampling analysis reveals the dynamics of the ETS.

**FIG 4:**
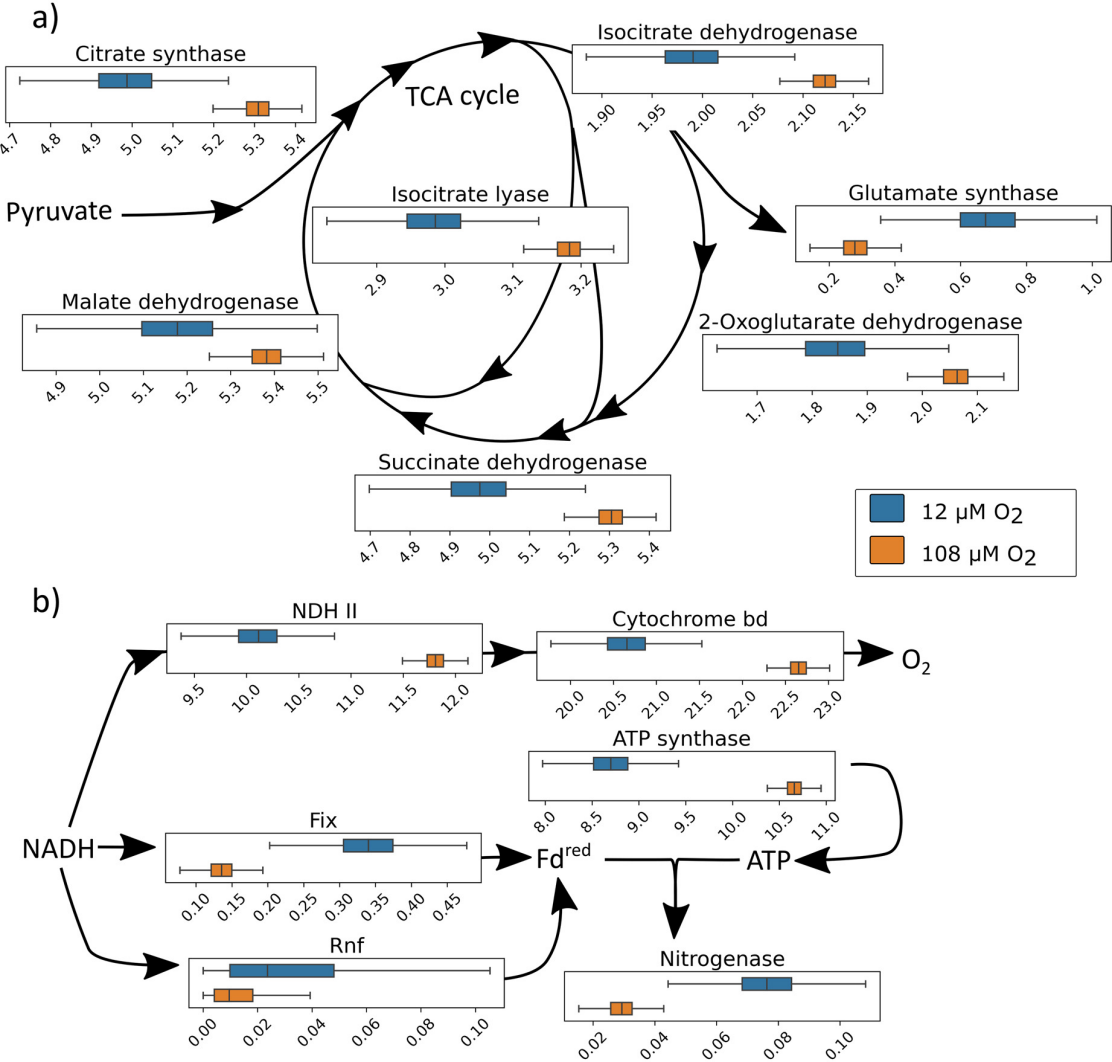
Histogram of flux samples normalized to sucrose uptake at different O_2_ concentrations. (a) Key NADH-producing enzymes of the TCA cycle show an increase of flux at higher [O_2_] (orange) than lower [O_2_] (blue). At the same time, electron-consuming reactions such as glutamate synthase are reduced at high [O_2_]. (b) The ETS shifts fluxes to accommodate [O_2_], where the partially coupled branch increases flux while electron flux to nitrogenase through ferredoxin (Fd^red^) is reduced. Flux is driven to oxygen reduction at the expense of nitrogen reduction. ATP generation is still maintained at a high rate to support NGAM in the model.

A flux sampling approach was taken to further understand the network’s variability under high- and low-O_2_ conditions. While similar in concept to flux variability analysis, flux sampling analysis provides a range of all feasible solutions and allows a distribution of the feasible fluxes, permitting statistics to be used in determining shifts of change between conditions (43). To assess the effect of increased O_2_ during nitrogen fixation, the maintenance constraints defined in Table 2 for 108 and 12 μM O_2_ were used. To be sure that the constraints produced models dependent only on O_2_ concentration, flux balance analysis (FBA) was used to show similar growth rates of 0.202 h^−1^ for 108 and 0.222 h^−1^ for 12 μM O_2_. Flux samples were taken and normalized to sucrose uptake rate to compare electron allocation in the model. NADH production in the TCA cycle increased in 108 μM O_2_ compared to 12 μM O_2_, but flux is decreased in NADH-consuming reactions such as those involving glutamate synthase, relative to carbon uptake (Fig. 4a). The partially coupled branch increases flux under higher O_2_ concentrations to protect nitrogenase and supply ATP for the increased maintenance rate. Electron transfer to nitrogenase through Rnf and Fix is reduced in higher O_2_ concentrations, while ATP synthase is increased overall, leading to less flux to nitrogenase (Fig. 4b).

### Efficient growth under metal-limited conditions.

A. vinelandii can adapt to the metal availability of its environment by using alternative nitrogenases. While the alternative nitrogenases use more common metals, such as V and Fe, they produce more H_2_ and are less efficient at reducing nitrogen than Mo-nitrogenase (equations 2 and 3) (3). This inefficiency of ammonia production acts as an unnecessary sink for electrons, reducing the growth rate of A. vinelandii under Mo-limited conditions. Interestingly, while the cost to fix nitrogen rises 30% for V-nitrogenase and 60% for Fe-only nitrogenase, growth rates do not show a complementary decrease (4, 44). This indicates compensation for the larger energy sinks of the alternative nitrogenase.

(2) $${\text{N}}_{2\;}+\;12e^{-}\;+\;\text{24MgATP}\;+\;{\text{12H}}^{+}\to 2{\text{NH}}_{3}\;+\;{\text{3H}}_{2}\;+\;\text{24MgADP}\;+\;{\text{16P}}_{\text{i}}$$

(3) $${\text{N}}_{2\;}+\;20e^{-}\;+\;\text{40MgATP}\;+\;{\text{20H}}^{+}\to {\text{2NH}}_{3}\;+\;{\text{7H}}_{2}\;+\;\text{40MgADP}\;+\;{\text{16P}}_{\text{i}}$$

To demonstrate growth under alternative nitrogenase conditions, the 108 μM O_2_ model was used, but the flux through Mo-nitrogenase or both Mo- and V-nitrogenase was set to zero for V- and Fe-only conditions, respectively. Growth rates were determined with FBA as well as O_2_ uptake rates and flux through each nitrogenase (Table 3). The model shows a slowing of growth by 13% for V conditions and 31% for Fe-only conditions, which follows but is not proportional to the increased cost of nitrogenase turnover. Additionally, only a small increase of flux to O_2_ consumption is required to maintain energy production in alternative conditions.

**TABLE 3:** Growth rates and nitrogenase rates decrease while respiration increase as the model switches from Mo- to V- to Fe-only nitrogenase^*a*^

Nitrogenase	Growth rate (h^−1^)	O_2_ consumption (mmol_O2_ · h^−1^ · g CDW^−1^)	Nitrogenase flux (mmol_N2_· h^−1^ · g CDW^−1^)
Mo	0.22	98.16	1.04
V	0.17	99.40	0.91
Fe	0.13	101.14	0.72

a All data were derived from the model with a sucrose uptake rate of 9 mmol_sucrose_/h/g CDW and an ATP maintenance rate of 110 mmol_ATP_/h/g CDW.

To further investigate the rearrangement of the A. vinelandii metabolism to compensate for alternative nitrogenase flux, the flux sampling method was used to determine probabilities for flux changes between conditions. Flux samples were plotted relative to the Mo-nitrogenase flux to determine the alternative nitrogenases’ positive or negative effect (Fig. S3). From the flux sampling, an increase in flux is seen through Fix, as the alternative nitrogenases are less efficient, requiring more reduced Fd. In addition, there is an increase of flux through uptake hydrogenase, which adapts to the increased hydrogen by-product. On the other hand, the flux through NDHII is decreased, even though overall respiration does not change as electron flux is compensated for by hydrogenase and Fix (Fig. S3).

10.1128/mBio.02593-21.6 FIG S3 Flux sampling of the alternative nitrogenase models. Flux samples were normalized to oxygen uptake rates. To highlight the shift from standard Mo-containing conditions, each flux sample was then normalized to its corresponding Mo flux. The dotted line was normalized to the Mo-containing condition flux (i.e., set at 1), so an increase in flux in other conditions is above this line or a decrease is below. Download FIG S3, TIF file, 1.7 MB. Copyright © 2021 Alleman et al. 2021 Alleman et al. https://creativecommons.org/licenses/by/4.0/ This content is distributed under the terms of the Creative Commons Attribution 4.0 International license.

### Optimal ammonia excretion under aerobic nitrogen-fixing conditions.

Unlocking A. vinelandii*’s* nitrogen fixation regulatory system by deleting the *nifL* gene allows nitrogenase to be constitutively expressed even in the presence of high ammonia concentrations in the medium (36, 45). The ability to engineer a robust ammonia-excreting strain has been a target for genetic engineering for many decades. By simulating the maximum ammonia production by A. vinelandii, key insights can be developed for future engineering targets for agricultural or industrial scenarios. To test the viability of ammonia excretion of the model and the effect of O_2_ maintenance, models of low and high O_2_ (12 and 108 μM O_2_) were set to excrete ammonia at a rate of 3 mmol_ammonia_ · h^−1^ · g CDW^−1^ as estimated from the work of Plunkett et al. (36). In these simulations, the increase of ammonia excretion essentially doubled the flux through nitrogenase and correspondingly reduced the growth rate (Table 4). Ammonia-excreting strains start to excrete ammonia within the stationary phase during batch growth (36). Cell growth would be minimal under these conditions, and O_2_ would be limited due to cell density. As maximal ammonia excretion starts in the early stationary phase, the predicted growth rate might not represent what is happening in the batch culture. Ammonia yields for high and low O_2_ are similar to the predicted 1.3 mol_sucrose_/mol_ammonia_, while ∼1.4 mol_sucrose_/mol_ammonia_ was experimentally determined. When O_2_ was increased to reduce the amount of time required for ammonia accumulation, an ammonia yield of ∼2.3 mol_sucrose_/mol_ammonia_ was experimentally determined, and 3 mol_sucrose_/mol_ammonia_ was predicted (36).

**TABLE 4:** Table of predicted fluxes for ammonia-assimilating, diazotrophic, and ammonia-excreting (Δ*nifL*) cells grown under high-oxygen (108 μM) and low-oxygen (12 μM) conditions^*a*^

Conditions	Growth rate (h^−1^)	Value (mmol of metabolite · h^−1^ · g CDW^−1^) for:			
		Ammonia exchange	Respiration rate	Sucrose uptake	Nitrogenase flux
Ammonia, high O_2_	0.30	−3.19	95.24	−9.00	0.00
Ammonia, low O_2_	0.33	−3.48	34.07	−4.00	0.00
Diazotrophic, high O_2_	0.20	0.00	98.02	−9.00	1.05
Diazotrophic, low O_2_	0.22	0.00	37.10	−4.00	1.15
Δ*nifL*, high O_2_	0.10	3.00	100.64	−9.00	2.04
Δ*nifL*, low O_2_	0.12	3.00	39.71	−4.00	2.14

a Negative exchange rates are uptakes into the cell, while positive values are excretions.

## DISCUSSION

The decoupling of energy consumption and biomass accumulation combined with an exceptionally high respiration rate in A. vinelandii cells led to the proposal of respiratory protection (46, 47, 68, 69). This proposal was reinforced with the discovery within A. vinelandii of a branch of ETS containing the uncoupled NADH dehydrogenase (NDHII) and the terminal oxidase cytochrome *bd* with high *V*_max_ (maximum reaction velocity) and low affinity for O_2_ (11, 22, 25, 41, 48). Others have disagreed with the basic principles of respiratory protection, as nitrogen fixation and O_2_ consumption are not correlated (16). The respiration rate plateaus after a concentration of 70 μM O_2_ with only a corresponding slight decrease of nitrogenase rate (15). While these observations of plateauing of O_2_ respiration are valid, the decoupling of energy from biomass still increases with O_2_ concentration. Inomura et al. developed a quantitative mechanistic model showing increased respiratory protection, including maintenance as the O_2_ concentration increases (33). The Inomura model relied on an energy transfer efficiency parameter to estimate the efficiency of the ETS to convert carbon into ATP. Using a stoichiometric model, a detailed understanding of flux through each reaction of the ETS can be predicted, allowing one to make an informed hypothesis and to develop engineering strategies.

Using experimental maintenance coefficients and the genome-scale model iAA1300, we have shown that the partially coupled branch of the ETS appears to be involved in metabolism at all measured O_2_ concentrations. Requiring the partially coupled branch was based on the need to factor the high *Azotobacter* NGAM into the model, which is very high compared to other genome-scale models of proteobacteria (49, 50). The minimization of NGAM is dependent on the decoupling of the ETS. However, transcript expression and spectrographic data suggest that the fully coupled branch may be active under normal nitrogen-fixing conditions (13, 23, 45, 51). Thus, both branches of the ETS may work together to balance diverse conditions. More significant energy dissipation through the NGAM mechanism would be required to model simultaneous use of both branches.

O_2_ reduction and energy production decoupling are not entirely accounted for by the partially coupled branch alone. The extra energy consumption required to maintain accurate growth is modeled as an ATP consumption reaction. This consumption most likely incorporates many different reactions and does not have to be ATP, but two categories can be proposed: (i) base metabolic reactions not accounted for in the model and (ii) reactions that respond to O_2_ and dissipate energy. For the first category, more accurate physiological data and biomass composition would help predict energetic needs. The current model predicts growth yields for 12, 44, and 108 μM O_2_ concentrations, so a significant change in the biomass equation is not expected (Fig. 4a). The A. vinelandii strain OP and derivatives such as strain DJ cannot produce alginate and do not produce poly(3-hydroxybutyrate) under high O_2_ and continuous culture (52–55). However, energy-consuming mechanisms like general protein turnover and unknown transport of metabolites or proteins might contribute to the basal NGAM. The second proposed category consists of reactions that respond to the O_2_ concentration and could be responsible for the energy dissipation. First, oxygen-dependent protein turnover and reactive O_2_ species in high O_2_ concentrations are unknown. Characterization of O_2_-sensitive A. vinelandii mutants showed that only three of 13 had a decreased respiration or catalase rate, leaving mechanisms other than respiratory protection as possibly responsible for O_2_ sensitivity (56). Also, cellular processes known to be active during nitrogen fixation are challenging to model in steady state, such as proton leakage, pilus formation, and the *in vivo* stoichiometry of nitrogenase (23, 57, 58). Additionally, other reactions can consume O_2_ with a low enough reduction potential, including Mehler reactions or soluble terminal oxidases (59, 60).

Preserving high NGAM through the partially coupled branch is required for growth under ammonia-supplemented conditions. While accurate data with high carbon and high ammonia are lacking, the use of diazotrophic maintenance rates closely predicted growth rates for the ammonia supplement model. Under high sucrose and O_2_ concentrations, ammonia-supplemented and nitrogen-fixing A. vinelandii strains respire at similar rates and offer similar steady-state protein levels (15), leading to the proposal that respiratory protection is not just a mechanism for nitrogenase protection but a core response to high carbon and O_2_ concentrations. The respiratory protection branch is regulated by CydR, an FNR regulatory protein that responds directly to O_2_ (25). The terminal oxidase cytochrome *bd* is not required for ammonia-supplemented growth (41). However, cytochrome *d*-deficient mutants grow poorly in ammonia-supplemented medium if not inoculated at high cell density, suggesting a role for cytochrome *d* in the higher-O_2_ environment of low-density cultures (41, 51). Using data from the Oelze lab, Inomura et al. proposed control of respiration and nitrogen fixation by the C/N ratio, in which excess substrate respiration increases with the C/N ratio until a point at which the C/N ratio is high enough for nitrogenase to be derepressed (34, 61). Although respiratory protection is more dependent on the C/N ratio than nitrogenase regulation, increased respiration at a high C/N ratio still secondarily protects nitrogenase from oxygen. While under both diazotrophic and ammonia-supplemented conditions, respiration plateaus above ∼70 μM O_2_ (15), we have shown that an increasing O_2_ concentration still requires high NGAM. The decoupling of energy and high NGAM in ammonia-supplemented growth could keep the cytosol in microaerobic or at a low redox potential to support oxygen-sensitive reactions unrelated to nitrogenase. Alternatively, it may be advantageous under high-carbon conditions to maintain low O_2_ to rapidly derepress nitrogenase in variable conditions.

To adapt to higher O_2_ concentrations, A. vinelandii must increase the availability of electrons. Flux sampling normalized to sucrose uptake shows an increased flux of NADH producing reactions of the TCA cycle and a decreased flux in other reactions, such as those with glutamate synthase, Fix, Rnf, and nitrogenase. As the flux to O_2_ reduction and ATP generation increases, the percent of energy allocated to nitrogen-fixing reactions decreases (Fig. S1). This explains why mutations in what should be beneficial enzymes such as Fix or Rnf and uptake hydrogenase do not affect growth under standard high O_2_ conditions, as the relative flux to nitrogenase is so small (24, 29, 62). Nevertheless, these reactions become more critical if more energy is allocated to nitrogenase under low-O_2_ or Mo-limited conditions. The increasing energy demand is significant under Mo-limited conditions, requiring 30% and 60% more energy for V-nitrogenase and Fe-only nitrogenase, respectively. Interestingly, under batch and continuous culture, A. vinelandii grows slightly more slowly in medium lacking Mo or lacking both Mo and V (44). The increased flux through hydrogenase and energy-conserving reactions like that with Fix allows A. vinelandii to maintain a higher growth rate. This general pattern shows when Rnf’s proton motive force mechanism is compared to Fix’s electron bifurcation mechanism. At lower O_2_ concentrations, the cell moves away from the energy-decoupling and respiration protection reactions and into maximizing energy production, favoring Fix, which supports the creation of proton motive force and ATP production. While kinetics and thermodynamics also influence the enzymes of the ETS, the stoichiometric pattern shows distinct roles for Rnf and Fix.

BNF can alleviate the cost and damage caused by industrial nitrogenous fertilizer. An ammonia-excreting strain of A. vinelandii can support plant growth and is a candidate for biofertilizer (63, 65, 66). Understanding the dynamics of metabolism under ammonia-excreting conditions will be essential to engineering more robust strains. Recent work optimized ammonia-excreting strains and showed 3 mmol_ammonia_ · h^−1^ · g CDW^−1^ excreted into the medium (36). Modeling these rates shows a doubling of flux through nitrogenase and a halving of growth rate. Experimentally, ammonia-excreting strains grow at rates similar to that of the wild type (WT), but the accumulation of ammonia in the medium occurs in the stationary phase during batch culture. This suggests that the WT is limited in the log phase by nitrogenase flux and that in the stationary phase, once carbon is low, nitrogenase becomes repressed. In contrast, ammonia-excreting strains are also nitrogenase limited in the exponential phase but cannot regulate nitrogenase in the stationary phase. More dynamic modeling of this phenomenon will allow for more optimization and balance, leading to a technology that will maximize ammonia yield.

### Conclusion.

We have been able to establish a genome-scale metabolic model of nitrogen fixation and adaptations to O_2_. This model gives a blueprint for future engineering strategies in nitrogen fixation and its ability to help offset nitrogenous fertilizer. We have shown that carbon concentration, O_2_ concentration, and ammonia supplementation affect the nitrogen fixation model. By adding the ETS to this model, we discovered that the contribution of respiratory protection, which previously was proposed to be a mechanism for diazotrophic conditions, might be a general response to high-carbon and high-O_2_ conditions. The cell compensates for allocating resources to an extraordinarily high maintenance rate by lowering growth yields and the rearrangement of the ETS. Future engineering in ammonia-excreting organisms must consider this balance between O_2_ reduction and nitrogen fixation and the complex relationship between the two.

## MATERIALS AND METHODS

### Model curation.

To build on the previous model, iDT1278 (35), essential reactions for diazotrophic growth were corrected for stoichiometry and annotation or added to the model (Table S1). See the supplemental methods in Text S1 for further model curation procedures.

10.1128/mBio.02593-21.8 TEXT S1 Supplemental materials and methods. Download Text S1, DOCX file, 0.02 MB. Copyright © 2021 Alleman et al. 2021 Alleman et al. https://creativecommons.org/licenses/by/4.0/ This content is distributed under the terms of the Creative Commons Attribution 4.0 International license.

### Maintenance rate quantification.

Maintenance coefficients are taken from Table 1 in the work of Kuhla and Oelze (39) and were converted to sucrose · h^−1^ · g CDW^−1^ using a ratio of 0.7 g protein/g CDW (15) and then used as the sucrose uptake rate for the model. Under these conditions, the assumption is that all energy goes to NGAM, causing a zero growth rate. Therefore, to determine the value of NGAM, the ATPM rate lower bound was increased until the growth reached zero, allowing all sucrose consumption to be allocated to NGAM. The determination of ATPM using this method depends on the ETS efficiency, so the fully coupled branch and the partially coupled branch of the ETS as well as the Rnf or Fix routes were used to determine a separated ATPM (Data Set S1). A specific ATPM flux was determined for each ETS branch under different O_2_ concentrations using the experimentally determined maintenance coefficient (Table 2).

### Model analysis and availability.

 See the supplemental methods (Text S1) for further model analysis procedures. All other data are available at https://github.com/alexander-alleman/Azotobactervinelandii_metabolicmodel (doi: 10.5281/zenodo.5184646). Metabolic models are saved in json and smbl format. All analysis and figure creation were documented in Jupyter notebooks.

10.1128/mBio.02593-21.3 TABLE S3 Basic model information and Memote criteria for the model presented in this paper (iAA1300) and the previous *A. vinelandii* model (iDT1278) (35) Table S3, XLSX file, 0.01 MB. Copyright © 2021 Alleman et al. 2021 Alleman et al. https://creativecommons.org/licenses/by/4.0/ This content is distributed under the terms of the Creative Commons Attribution 4.0 International license.
