# Supplementary figures and images for: Metabolic Model of the Nitrogen-Fixing Obligate Aerobe Azotobacter vinelandii Predicts Its Adaptation to Oxygen Concentration and Metal Availability
Source: mBio. 2021 Dec 14;12(6):e02593-21. doi: 10.1128/mBio.02593-21 (PMC8686835; doi:10.1128/mBio.02593-21)

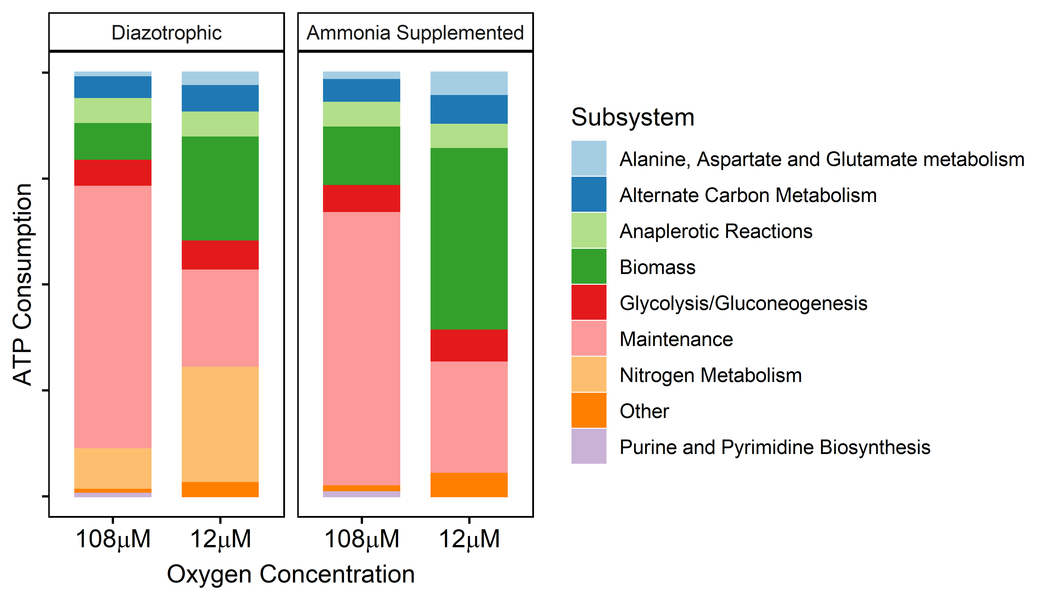

Supplement: FIG S1 [file mbio.02593-21-sf001.tif]

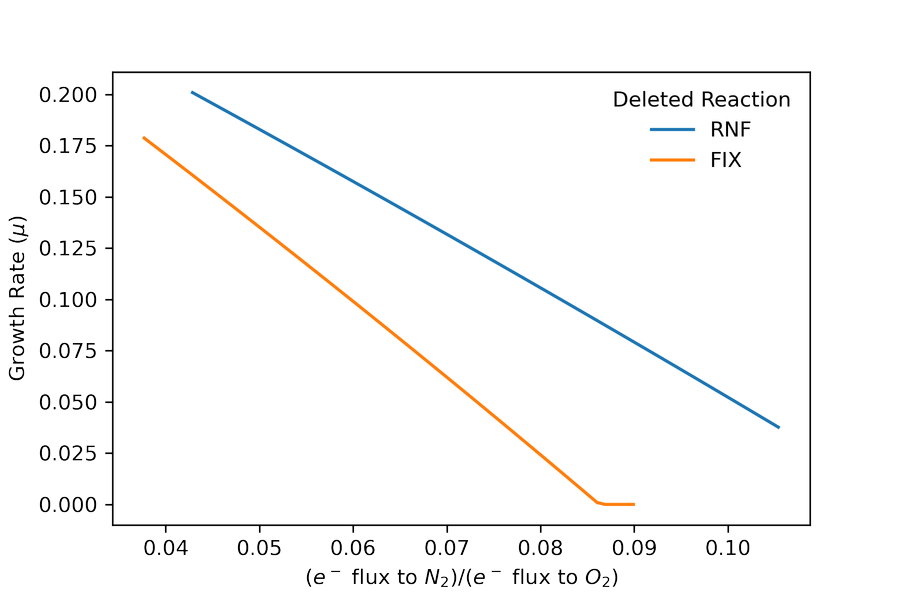

Supplement: FIG S2 [file mbio.02593-21-sf002.tif]

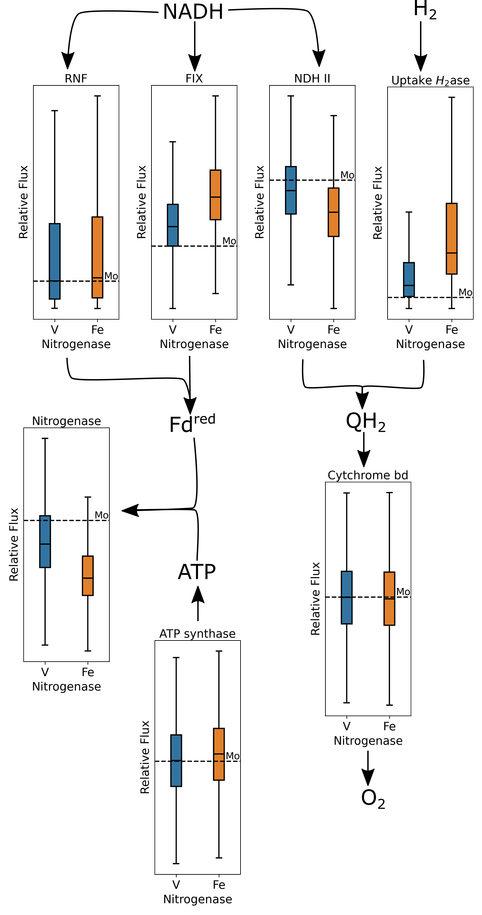

Supplement: FIG S3 [file mbio.02593-21-sf003.tif]
