# Supplementary material for: Metabolic Model of the Nitrogen-Fixing Obligate Aerobe Azotobacter vinelandii Predicts Its Adaptation to Oxygen Concentration and Metal Availability
Source: mBio. 2021 Dec 14;12(6):e02593-21. doi: 10.1128/mBio.02593-21 (PMC8686835; doi:10.1128/mBio.02593-21)
Supplement: TEXT S1 [file mbio.02593-21-s0001.docx]

Supplemental Materials and Methods

**Model curation**- Enzymes of the ETS were added to the *iAA1300* model, including the electron bifurcating Fix complex, fully coupled NADH dehydrogenase I complex, cytochrome c oxidoreductase, nitrogenase homologs V-nitrogenase and Fe-only nitrogenase, as well as a soluble hydrogenase and a transhydrogenase. Other reactions were either reannotated or removed. All reactions using menaquinone were removed as *A. vinelandii* only contains quinone (1–3). Glucose uptake was constrained to reaction GLCt2pp (*gluP*) (4). The Rnf reaction stoichiometry was changed from 3 protons translocated to 6 protons translocated based on thermodynamic and kinetic analysis as found at the connected GitHub page in the RNF_stoich.ipynb jupyter notebook. The ED pathway and the glyoxylate shunt were constrained to ratios determined by metabolic flux analysis (5) using a custom Python function based on COBRA MatLab function addRatioReaction (6).

The model was cleared of dead-end reactions and orphaned metabolites while maintaining genome-relevant reactions. Out of the 2,289 reactions, 278 were essential, while 928 were categorized as blocked reactions that could not carry flux. To allow the model to be built upon in the future, reactions with a corresponding gene are kept even if the reactions are blocked. Of the blocked reactions, 177 had no associated genes and were removed as they are not involved in gap-filling or gene homology. Following the removal of blocked “geneless” reactions, an additional 45 metabolites were also removed.

For annotation standard consistency with model *iAA1300*, the Memote software was used with summary statistics reported in Table S3 and fully reported on the GitHub page (7).

***Flux balance analysis-*** All calculations were done with the cobrapy 0.21.0 (8). For flux balance analysis, the optimization problem is formulated as:

$$\max Z$$

$$given:$$

$$\left\{ \begin{aligned} S \cdot v=0 \\ b_{i}\leq v_{i}\leq c_{i} \\ Z= \sum_{k} n_{k}v_{k} \end{aligned} \forall v_{i}\in v \right.$$

With Z being the biomass equation, with the stoichiometric coefficients $n_{k}$ and the biomass flux as $v_{k}$. Biomass coefficients have a unit of hr^-1^ and represent the specific growth rate. $S$ represents the stoichiometry matrix, and $v$ is the flux vector. The scalars $b_{i}$ and $c_{i}$ are the lower and upper bounds for each flux $v_{i}$.

**Predicting growth with maintenance rates** -Testing the ATPM/NGAM values was done by setting the ATPM rate within the model and then increasing the sucrose uptake rate to the experimentally derived value found in Figure 4 of Kuhla and Oelze (9). Figure data points were taken using WebPlotDigitizer version 4.3 (10). With experimentally determined sucrose uptake rate and theoretically determined ATPM rates, a growth rate was predicted. The predicted growth rates were plotted against the known growth rates for both the fully-coupled and partially-coupled branches of the ETS. Determining mean standard error (MSE), mean absolute error (MAE), and root mean squared error (RSME) were all measured using the package scipy.stats (11).

***Flux sampling****-* Flux sampling analysis was conducted in COBRApy (8) using the optGpSampler (12) algorithm using 100000 samples with a thinning rate of 10000 in accordance with Hermann et al. (13). Model constraints for flux sampling were used from previous analyses for 108 µM and 12 µM O_2_ with the experimentally derived sucrose uptake rates of 9 and 4 mmol of sucrose ∙ hr^-1^ ∙ gCDW^-1^, respectively. In addition, the maintenance rates were used from the previous analyses of 110 mmol of ATP ∙ hr^-1^ ∙ gCDW^-1^ for 108 µM O_2_ and 16 mmol of ATP ∙ hr^-1^ ∙ gCDW^-1^ for 12 µM O_2_. Traditional FBA analyses were also performed to compare sampling analyses showing growth rates of 0.202 hr^-1^ and 0.222 hr^-1^ for 108 µM and 12 µM O_2_, respectively. All plots were made in Python using Matplotlib.

***Ammonia excretion****-* The ammonia excreting model was modified from the glucose model based on constraints of Wu et al. (5) and ATPM rates determined above. The model was first simulated for average growth under experimental conditions with an excretion rate of 3 mmol_Ammonia_ ∙ hr^-1^ ∙ g CDW^-1^ determined in Plunkett et al. (14).

***Alternative nitrogenases****-* The alternative nitrogenase enzymes of V-nitrogenase and Fe-only nitrogenase were simulated for growth and flux sampling under standard sucrose conditions of 9 mmol of sucrose ∙ hr^-1^ ∙ gCDW^-1^ and an ATPM of 110 mmol of ATP ∙ hr^-1^ ∙ gCDW^-1^. While these conditions are not experimentally determined for alternative growth, they are a close approximation for batch growth cultures under high O_2_ and carbon but metal limited conditions. To determine growth rates, FBA was used, and flux sampling was conducted as stated above.

References

1. Wong T-Y, Maier RJ. 1984. Hydrogen-Oxidizing Electron Transport Components in Nitrogen- Fixing *Azotobacter vinelandii*. J Bacteriol 159:5.

2. Jurtshuk P, Bednarz AJ, Zey P, Denton CH. 1969. L-malate oxidation by the electron transport fraction of *Azotobacter vinelandii*. J Bacteriol 98:1120–1127.

3. Jones CW, Redfearn E. Electron Transport in *Azotobacter vinelandii*. Biochimica et Biophysica Acta 113:467–481.

4. Quiroz-Rocha E, Moreno R, Hernández-Ortíz A, Fragoso-Jiménez JC, Muriel-Millán LF, Guzmán J, Espín G, Rojo F, Núñez C. 2017. Glucose uptake in Azotobacter vinelandii occurs through a GluP transporter that is under the control of the CbrA/CbrB and Hfq-Crc systems. 1. Sci Rep 7:858.

5. Wu C, Herold RA, Knoshaug EP, Wang B, Xiong W, Laurens LML. 2019. Fluxomic Analysis Reveals Central Carbon Metabolism Adaptation for Diazotroph *Azotobacter vinelandii* Ammonium Excretion. 1. Sci Rep 9:13209.

6. Heirendt L, Arreckx S, Pfau T, Mendoza SN, Richelle A, Heinken A, Haraldsdóttir HS, Wachowiak J, Keating SM, Vlasov V, Magnusdóttir S, Ng CY, Preciat G, Žagare A, Chan SHJ, Aurich MK, Clancy CM, Modamio J, Sauls JT, Noronha A, Bordbar A, Cousins B, El Assal DC, Valcarcel LV, Apaolaza I, Ghaderi S, Ahookhosh M, Ben Guebila M, Kostromins A, Sompairac N, Le HM, Ma D, Sun Y, Wang L, Yurkovich JT, Oliveira MAP, Vuong PT, El Assal LP, Kuperstein I, Zinovyev A, Hinton HS, Bryant WA, Aragón Artacho FJ, Planes FJ, Stalidzans E, Maass A, Vempala S, Hucka M, Saunders MA, Maranas CD, Lewis NE, Sauter T, Palsson BØ, Thiele I, Fleming RMT. 2019. Creation and analysis of biochemical constraint-based models using the COBRA Toolbox v.3.0. 3. Nat Protoc 14:639–702.

7. Lieven C, Beber ME, Olivier BG, Bergmann FT, Ataman M, Babaei P, Bartell JA, Blank LM, Chauhan S, Correia K, Diener C, Dräger A, Ebert BE, Edirisinghe JN, Faria JP, Feist AM, Fengos G, Fleming RMT, García-Jiménez B, Hatzimanikatis V, van Helvoirt W, Henry CS, Hermjakob H, Herrgård MJ, Kaafarani A, Kim HU, King Z, Klamt S, Klipp E, Koehorst JJ, König M, Lakshmanan M, Lee D-Y, Lee SY, Lee S, Lewis NE, Liu F, Ma H, Machado D, Mahadevan R, Maia P, Mardinoglu A, Medlock GL, Monk JM, Nielsen J, Nielsen LK, Nogales J, Nookaew I, Palsson BO, Papin JA, Patil KR, Poolman M, Price ND, Resendis-Antonio O, Richelle A, Rocha I, Sánchez BJ, Schaap PJ, Malik Sheriff RS, Shoaie S, Sonnenschein N, Teusink B, Vilaça P, Vik JO, Wodke JAH, Xavier JC, Yuan Q, Zakhartsev M, Zhang C. 2020. MEMOTE for standardized genome-scale metabolic model testing. 3. Nat Biotechnol 38:272–276.

8. Ebrahim A, Lerman JA, Palsson BO, Hyduke DR. 2013. COBRApy: COnstraints-Based Reconstruction and Analysis for Python. BMC Syst Biol 7:74.

9. Kuhla J, Oelze J. 1988. Dependency of growth yield, maintenance and Ks-values on the dissolved oxygen concentration in continuous cultures of *Azotobacter vinelandii*. Arch Microbiol 149:509–514.

10. Rohatgi A. WebPlotDigitizer User Manual Version 4.3 23.

11. Virtanen P, Gommers R, Oliphant TE, Haberland M, Reddy T, Cournapeau D, Burovski E, Peterson P, Weckesser W, Bright J, van der Walt SJ, Brett M, Wilson J, Millman KJ, Mayorov N, Nelson ARJ, Jones E, Kern R, Larson E, Carey CJ, Polat İ, Feng Y, Moore EW, VanderPlas J, Laxalde D, Perktold J, Cimrman R, Henriksen I, Quintero EA, Harris CR, Archibald AM, Ribeiro AH, Pedregosa F, van Mulbregt P. 2020. SciPy 1.0: fundamental algorithms for scientific computing in Python. 3. Nature Methods 17:261–272.

12. Megchelenbrink W, Huynen M, Marchiori E. 2014. optGpSampler: An Improved Tool for Uniformly Sampling the Solution-Space of Genome-Scale Metabolic Networks. PLOS ONE 9:e86587.

13. Herrmann HA, Dyson BC, Vass L, Johnson GN, Schwartz J-M. 2019. Flux sampling is a powerful tool to study metabolism under changing environmental conditions. 1. NPJ Syst Biol Appl 5:1–8.

14. Plunkett MH, Knutson CM, Barney BM. 2020. Key factors affecting ammonium production by an *Azotobacter vinelandii* strain deregulated for biological nitrogen fixation. Microbial Cell Factories 19:107.
